# HIF-1α Induces Multidrug Resistance in Gastric Cancer Cells by Inducing MiR-27a

**DOI:** 10.1371/journal.pone.0132746

**Published:** 2015-08-20

**Authors:** Qun Zhao, Yong Li, Bi-bo Tan, Li-qiao Fan, Pei-gang Yang, Yuan Tian

**Affiliations:** Department of General Surgery, the Fourth Affiliated Hospital, Hebei Medical University, Shijiazhuang, China; H.Lee Moffitt Cancer Center & Research Institute, UNITED STATES

## Abstract

This study aimed to determine the correlation between HIF-1α and miR-27a expression and to evaluate the effect of inhibition of HIF-1α expression on miR-27a expression and drug resistance in gastric cancer (GC). In the present study, real time-PCR and Western blot were performed to detect the expression of HIF-1α in GC tissues and cell lines. Then, OCUM-2MD3/L-OHP cells were transfected with HIF-1α-siRNA, a miR-27a mimic or pcDNA-HIF-1α, and cell survival was determined via the MTT assay. The expression of HIF-1α, miR-27a, and MDR-related genes was measured via real time-PCR and Western blot. ChIP and dual luciferase activity assays were performed to assess the transcriptional regulation of HIF-1α and miR-27a. The results revealed that transfection with HIF-1α-siRNA markedly decreased the levels of miR-27a, resulting in dramatically enhanced inhibition of the proliferation rate of OCUM-2MD3/L-OHP cells. Compared to non-transfected cells, the survival rate was significantly reduced in the cells transfected with HIF-1α-siRNA after treatment with L-OHP. The cell survival rate was significantly increased in OCUM-2MD3/L-OHP cells transfected with the miR-27a mimic, whereas HIF-1α overexpression did not result in any clear change in cell survival. The results of the dual luciferase activity assay demonstrated that HIF-1α enhances the transcriptional activity of the miR27a promoter in cells transfected with a reporter plasmid containing the upstream promoter region of miR27a together with pcDNA-HIF-1α. ChIP analysis suggested that HIF-1α directly binds to the promoter region of miR27a. Inhibition of HIF-1α or miR27a expression decreased MDR1/P-gp, LRP, and Bcl-2 expression in OCUM-2MD3/L-OHP cells. Thus, we found that HIF-1α is closely associated with MDR in GC and that HIF-1α may suppress MDR1/P-gp, LRP and Bcl-2 expression by inhibiting miR-27a expression.

## Introduction

Gastric cancer (GC) is among the most common malignancies, causing serious harm worldwide [1, 2] After years of technological advances in the diagnosis and treatment of GC, its incidence and mortality have declined worldwide but remain high in Asian countries [3, 4]. Currently, gastric resection is the only available method to cure GC. However, it is difficult to achieve a complete cure despite surgical removal of the tumor because most patients suffer from advanced GC upon diagnosis [5, 6]. Therefore, chemotherapy plays an extremely important role in the comprehensive treatment of GC. Although chemotherapy greatly progressed with respect to the treatment of advanced GC, [7, 8] the prognosis of GC remains inadequate, with a 5-year survival rate of less than 30% [9]. This prognosis is primarily due to the multidrug resistance (MDR) of GC cells. MDR in GC often leads to the failure of chemotherapy [10–12]. Therefore, there is an urgent need to develop novel promising therapeutic strategies to effectively reduce MDR in GC.

Oxygen deficiency is prevalent in solid tumors and is associated with a variety of biological functions. Currently, hypoxia-inducible factor (HIF)-1α is considered to be closely associated with hypoxia. HIF-1α is strongly expressed in a variety of malignant tumors [13, 14] and acts as an essential factor to regulate the adaption of tumor cells to hypoxia [15]. HIF-1α has been suggested to be closely associated with GC MDR [16, 17]. However, it is unclear which pathway mediates the role of HIF-1α in GC MDR.

In recent years, the role of microRNAs (miRNAs) in cancer has become a widely investigated mechanism of tumor initiation and treatment. miR-27a, a member of the miRNA family, has been shown to affect the MDR of GC [18]. Furthermore, the expression of miR-27a is increased in a hypoxic environment [19]. These findings suggest that HIF-1α may regulate the expression of miR-27a and affect GC MDR. However, the specific regulatory mechanisms have yet to be elucidated. The present study showed that the expression of HIF-1α and miR-27a were significantly up-regulated in GC tissues and cell lines, especially in resistant cell lines.

Transfection with a specific small interfering RNA to block endogenous HIF-1α resulted in a reduction in miR-27a expression and the alleviation of MDR in GC cell lines. These novel findings suggest that inhibition of HIF-1α expression suppresses the transcription of the MDR-related genes MDR1/P-gp, LRP, and Bcl-2 to attenuate MDR of GC cells by repressing miR-27a expression.

## Materials and Methods

### 1.1 Materials

Gastric cell line OCUM-2MD3 was from Professor Masakazu Yashiro in Japan Oita Medical Surgery[20]. The stable drug-resistant cell line OCUM-2MD3/L-OHP2 was obtained via culturing and selection by our research group. The GSE-1 cell line was purchased from the Cell Resource Center at Shanghai Institutes for Biological Sciences of the Chinese Academy of Sciences. RPMI 1640 culture medium and trypsin were purchased from Gibco Company; Trizol reagent and Lipofectamine 2000 transfection reagent were purchased from Invitrogen. The reverse transcription kit and fluorescence quantitative PCR reagents were obtained from Promega Corporation. PCR primers and small interfering RNA were synthesized by Shanghai Biological Engineering Company. The protein extraction kit was obtained from Beyotime Company, China. Primary antibodies against HIF-1α, MDR1/P-gp, GST-π, LRP, Bcl-2, TS or GAPDH were purchased from Santa Cruz. MTT was obtained from Sigma. Our study was approved by the ethics committee of the Fourth Affiliated Hospital of Hebei Medical University.

### 1.2 Clinical sample preparation

All 65 patients with GC were selected after gastric resection and pathological confirmation in the Fourth Hospital of Hebei Medical University, including 42 males and 23 females aged 60.5±8.1 years who had not received preoperative radiotherapy or chemotherapy. Cancer and para-carcinoma tissues (approximately 1.0 cm×0.5 cm×0.5 cm) were taken from every patient, and the specimens were rapidly frozen in liquid nitrogen and subsequently transferred to -80℃ conservation. All participants signed the informed consent.

### 1.3 Cell culture and transfection

The OCUM-2MD3, OCUM-2MD3/L-OHP and GSE-1 cell lines were cultured in RPMI 1640 medium supplemented with 10% fetal bovine serum (FBS), 100U/ml penicillin and 100 mg/ml streptomycin. Notably, the medium of the OCUM-2MD3/L-OHP cells was treated with L-OHP at a concentration of 75μg/mL to maintain their drug-resistance phenotype, one week before surgery to stop the treatment. The cells were incubated in a humidified 5% CO_2_ atmosphere at 37°C.

Three HIF-1α-siRNA sequences were designed using BLOCK-iT RNAi Designer (sequence 1: GAGGAAACUUCUGGAUGCUGGUGAUtt; sequence 2: GGAUGCUGGUGAUUUGGAUAUUGAAtt; sequence 3: CAGGACAGUACAGGAUGCUUGCCAAtt), each of which was annealed with their complementary sequence and transfected respectively into the OCUM-2 MD3/L-OHP GC cells. A non-specific siRNA sequence (NS-siRNA: GAGUGGGUCUGGGUCUUCCCGUAGAtt) was used as a negative control. The miR27a mimetic sequence was 5'-UUCACAGUGGCUAAGUUCCGC-3 '. Human full-length HIF-1α coden sequence was subcloned into pcDNA3.1 vector. pGL3-miR27a-luc, pcDNA-HIF-1α, pGL3-Basic and pRL-TK plasmids were constructed and conserved in our laboratory.

The GC cells were seeded in 6-well plates for 24 h before transfection at a density of 4×10^5^ cells/ml. The plasmid vectors, siRNA or miR27a mimic was transfected into the GC cells or drug-resistant cells using the transfection reagent Lipofectamine following the manufacturer’s instructions. Then, the cells were washed with serum-free RPMI 1640 lacking antibiotics. The transfection efficiency was measured 24 h later, followed by the subsequent experiments.

### 1.4 MTT assay

GC tissues and normal para-carcinoma tissue was homogenized to prepare single cell suspensions by filtering through a 300-mesh copper grid. The GC cells digested using 0.02% EDTA-0.25% trypsin were seeded at a density of 5×10^4^ cells/ml in 96-well plates. Once the cells reached about 60% confluence, HIF-1α-siRNA was transfected or a chemotherapeutic drug (150μg/ml of L-OHP) was applied. Each group consisted of six wells. Then, 20μl of 5 mg/ml MTT was added for 4 h before the end of the experiment. The cells were cultured for 4 h, and then, the culture medium was discarded. Next, 150μl of DMSO was added to each well, and the OD values were measured at 490 nm using a microplate reader after shaking the plate for 15 min at room temperature. Experiments were repeated three times.

### 1.5 RNA isolation and quantitative RT-PCR

Total RNA was extracted using Trizol one-step method, and 2μg RNA was used for reverse transcription to generate template cDNA. The relative mRNA levels were determined via quantitative PCR, GAPDH served as an internal reference gene. The PCR parameters were as follow: 95°C for 5 min followed by 45 cycles of denaturation at 94°C for 30 s and annealing at 60°C for 30 s. The primer sequences were designed using Primer 5.0 and were searched for specificity. The primer sequences are as follows: HIF-1α (93 bp): (F) 5'-GACAGCCTCACCAAACAGAG-3' and (R) 5'-CTCAAAGCGACAGATAACACG-3'; MDR1(126 bp): (F) 5'-GAATGTTCAGTGGCTCCGAG-3' and (R) 5'-ACAATCTCTTCCTGTGACACC-3'; GST-π(151 bp): (F) 5'-ATACCATCCTGCGTCACCTG-3' and (R) 5'-TCCTTGCCCGCCTCATAGTT-3'; Bcl-2 (98 bp): (F) 5'-TGTGTGGAGAGCGTCAACC-3' and (R) 5'-TGGATCCAGGTGTGCAGGT-3'; LRP (129 bp): (F) 5'-TTTCTGACGGCAACTTCAAC-3' and (R) 5'-AGTCCAATGTCCAGCCCAT -3'; TS(129 bp): (F) 5'-TTTCTGACGGCAACTTCAAC-3 ' and (R) 5'-AGTCCAATGTCCAGCCCAT -3'; and GAPDH(138bp): (F) 5'-GACCCCTTCATTGACCTCAAC-3' and (R) 5'-CGCTCCTGGAAGATGGTGAT-3'. The quantitative PCR results were calculated using the 2^-ΔΔCt^ method.

### 1.6 Western blot

The tissue and cell samples were lysed using RIPA lysis buffer: 1% Triton X-100, 150 mM NaCl, 10 mM Tris-HCl, pH 7.4, 1 mM EDTA, 1 mM EGTA, pH 8.0, 0.2 mM Na3VO4, 0.2 mM phenylmethylsulfonyl fluoride, and 0.5% NP-40. Equal amounts of the protein samples were separated on the 10% polyacrylamide SDS gels (SDS-PAGE) and were electrotransferred to a polyvinylidene fluoride (PVDF) membrane (Amersham Pharmacia Biotech). The membranes were blocked with 5% BSA for 2 h and incubated with the primary antibody overnight at 4°C. The membranes were incubated for 2 h in a horseradish peroxidase-conjugated secondary antibody. The target bands were detected using an enhanced chemiluminescence (ECL) detection kit (Santa Cruz, USA). β-actin was used as an endogenous control gene. The experiment was replicated three times.

### 1.7 Chromatin immunoprecipitation (ChIP) assay

ChIP assays were performed according to the method from Wang et al. [21]. Cells with different treatment were fixed with 1% formaldehyde for 15 min at room temperature, and terminated by a final concentration of 0.125 M glycine. Then cells were lysed using 300μl lysis buffer (50 mM Tris-HCl, pH 8.0, 150 mM NaCl, 5 mM EDTA, 1% Nonidet P-40, 0.5% deoxycholate, and protease inhibitors). The cell lysates were sonicated in ice water bath to yield chromatin fragments about 600 bp, as assessed by agarose gel electrophoresis. After centrifugation at 13,000 rpm for 10 min, the supernatants were taken and pre-cleared for 15 min at 4°C via incubation with 30μl of protein A-Sepharose beads and sheared salmon sperm DNA. After centrifugation at 13,000 rpm for 5 min, the supernatants were divided into three equal parts: one for input, the other two for immunoprecipitation with or without HIF-1α antibody. The next day, the immune complexes were precipitated with protein A-Sepharose beads and sheared salmon sperm DNA, then the beads were collected after washed twice with the wash buffer I (20 mM Tris-HCl, pH 8.1, 150 mM NaCl, 0.1% SDS, 1% Triton X-100, and 2 mM EDTA), followed by wash buffer II (20 mM Tris-HCl, pH 8.1, 500 mM NaCl, 0.1% SDS, 1% Triton X-100, and 2 mM EDTA), and wash buffer III (10 mM Tris-HCl, pH 8.1, 0.25 M LiCl, 1% Nonidet P-40, 1% deoxycholate, and 1 mM EDTA), and the final wash buffer IV (10 mM Tris-HCl, pH 8.1, and 1 mM EDTA). The immunoprecipitates were eluted by 200μl elution buffer (1% SDS and 0.1 M NaHCO_3_), followed by incubation at 65°C overnight. The next day, DNA of each sample was isolated, and PCR was performed to amplify the promoter segments containing a HIF-1α binding site.

### 1.8 Luciferase assays

Cells grown to 70% confluence and then were transfected in triplicate with pGL3-miR-27a-luc, pcDNA-HIF-1α or pGL3-Basic, along with pRL-TK. After 48 h of transfection, cells were collected and the luciferase activity was measured using the Dual-Luciferase Reporter Assay System (Promega, Madison, WI) according to the manufacturer’s protocol. The luciferase activities of pRL-TK were served as internal control.

### 1.9 Statistical analysis

The results are presented as the means ± S.D. ANOVA, and Dunnett’s test was performed using SPSS 11.5 software.

## Results

### 1 HIF-1α and miR27a are differentially expressed between gastric para-carcinoma tissue and GC tissue

**Fig 1:**
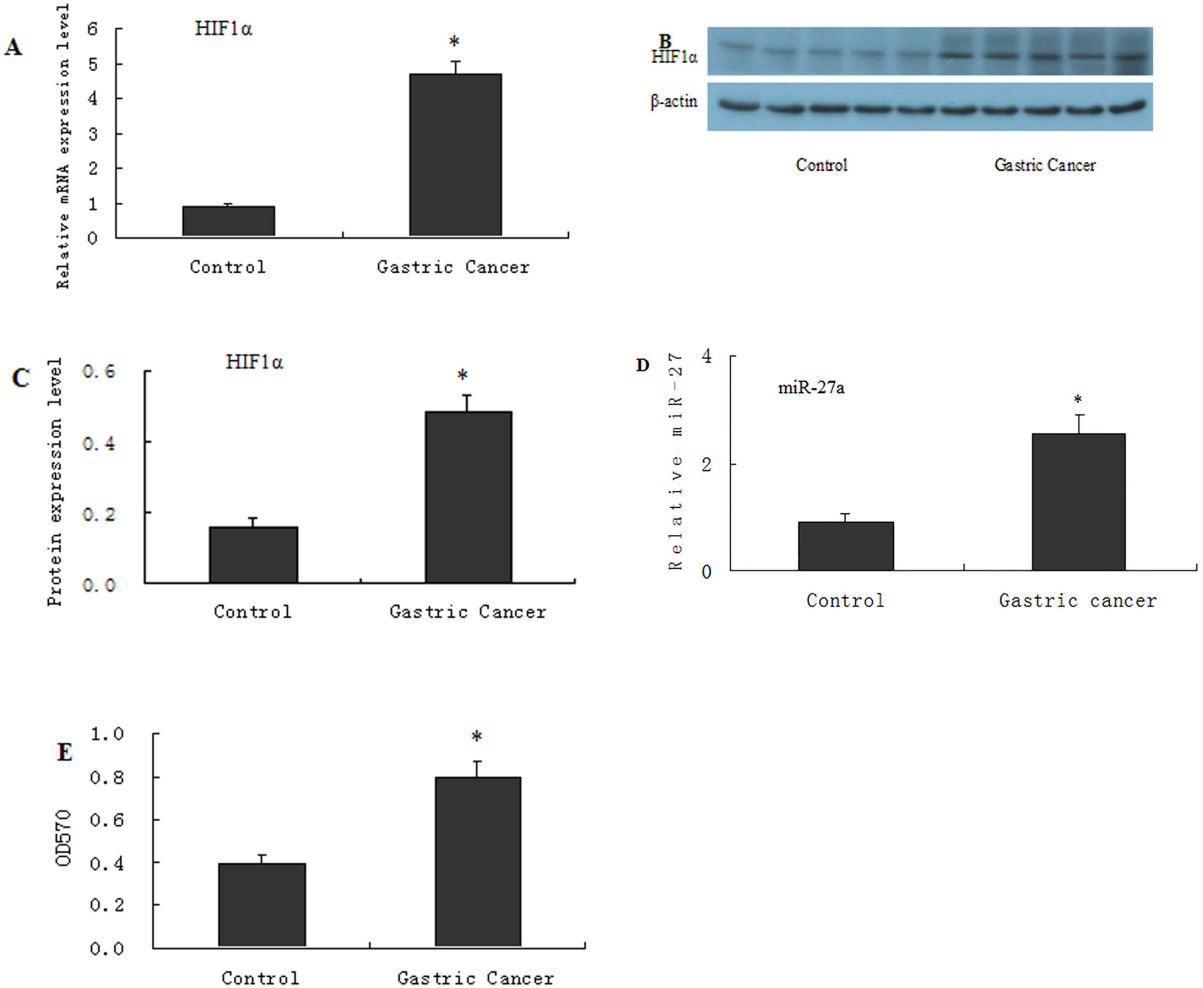
HIF1α and miR27a are highly expressed in gastric cancer tissues. Clinical gastric tumor specimens as well as normal control tissues were obtained and subjected to RT-qPCR (A) and Western blotting (B and C) to determine the expression of HIF1α, miR-27a was determined by QPCR (D). For RT-qPCR and Western blotting assays,β-actin was used for an endogenous reference to standardize the mRNA and protein expression levels. Single-cell suspension was made from the cancer tissues and the cancer-adjacent normal tissues and cultured for MTT assay. The cells were treated with chemotherapeutics (L-OHP, 150μg/mL) when they reached 80% confluence and used for MTT assay (E). 6 duplicates for each treatment were performed. Results were presented as the mean ± S.D. (n = 3). *P<0.01 compared with cancer-adjacent normal tissues group.

The expression of HIF-1α in GC tissue compared with gastric para-carcinoma tissue was determined via qRT-PCR and Western blot, and the sensitivity of L-OHP cells was determined via the MTT assay. HIF-1α (Fig 1A, 1B and 1C, qPCR and Western blot) and miR27a (Fig 1D, qPCR results) were up-regulated in GC tissue compared to gastric para-carcinoma tissue. The MTT assay demonstrated that the cell survival rate was greater in GC tissue than in gastric para-carcinoma tissue when L-OHP was added to the single-cell tissue suspensions (Fig 1E, histogram results).

### 2 HIF-1α and miR27a are differentially expressed between a gastric mucosa epithelial cell line and a GC cell line

**Fig 2:**
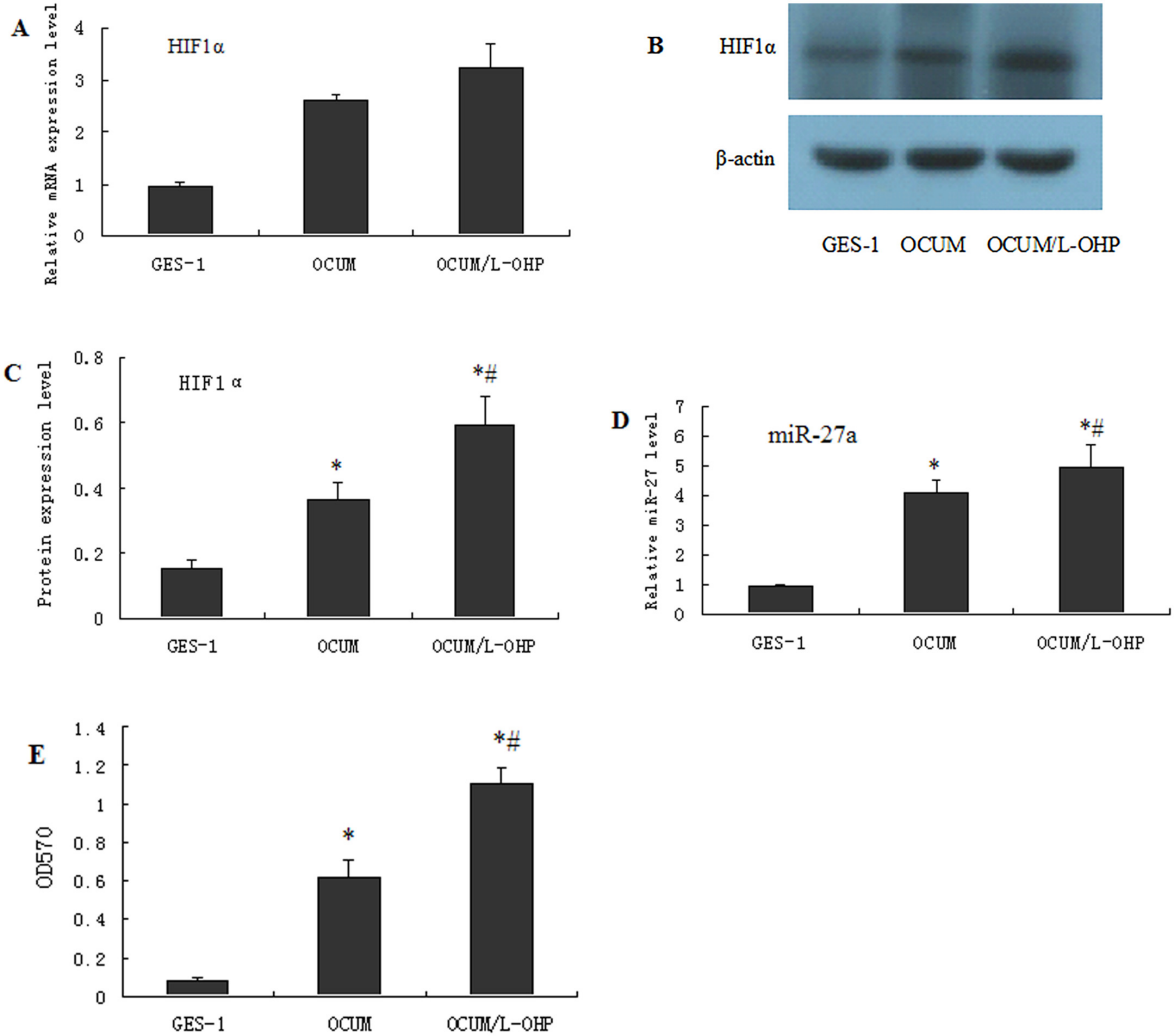
HIF-1α and miR27a are differently expressed in gastric mucosa epithelial cell line and gastric cancer cell line. Normal control human gastric mucosa epithelial cell line GES-1 as well as gastric cancer cell lines OCUM-2MD3 and OCUM-2MD3/L-OHP were subjected to RT-QPCR (A) and Western blotting (B and C) to compare the expression of HIF1α in vitro. miR-27a was determined by QPCR (D).For RT-QPCR and Western blotting assays, β-actin was used as an endogenous control to standardize the mRNA and protein expression levels. MTT assay was used to determine the survival rate of gastric cancer cell lines after the treatment with L-OHP in the cells of OCUM-2MD3/L-OHP, OCUM-2MD3 and GSE-1 (E). Results were presented as the mean ± S.D. (n = 3). *P<0.05 compared with cancer-adjacent normal tissues or GES-1 cells. The images shown are representatives of at least three independent experiments.

The expression of HIF-1α was the highest in OCUM-2MD3/L-OHP and OCUM-2MD3 cells, sequentially, and was the lowest in GES-1 cells (Fig 2A, 2B and 2C, qPCR and Western blot). Moreover, the expression of miR27a displayed the same trend, in which OCUM-2MD3/L-OHP cells displayed the highest expression, followed by OCUM-2MD3 cells, and GSE-1 cells displayed the lowest expression of miR-27a (Fig 2D, qPCR results). The MTT assay revealed that when L-OHP was added to the three cell lines, OCUM-2MD3/L-OHP cells exhibited the highest cell survival rate, followed by OCUM-2MD3 cells, and that GSE-1 cells exhibited the lowest cell survival rate (Fig 2E, histogram results).

### 3 HIF-1α-siRNA suppresses the expression of miR27a and the drug resistance of OCUM-2MD3/L-OHP cells

**Fig 3:**
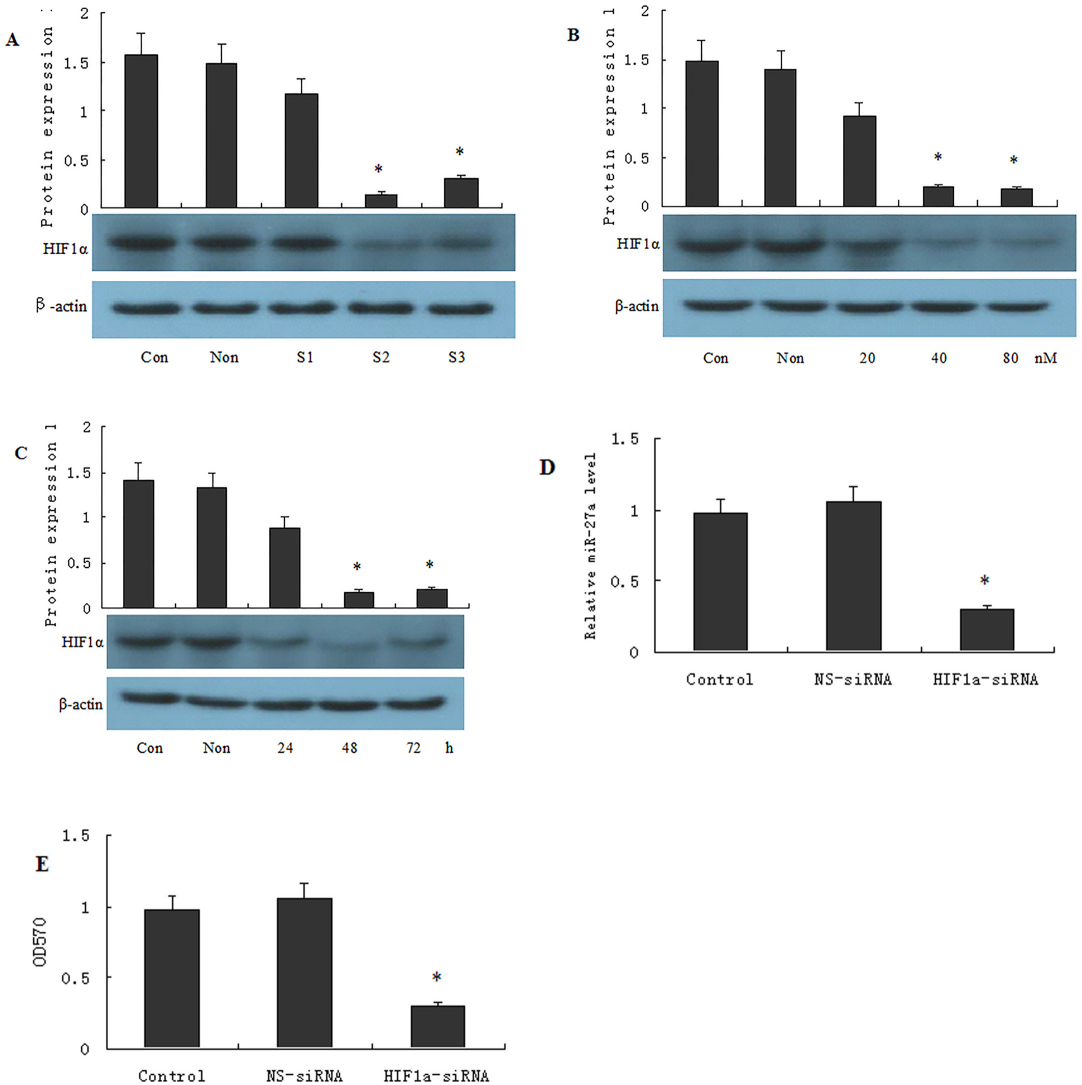
Inhibition of HIFαusing siRNA suppresses expression levels of miR27a and reduces drug resistance of OCUM-2MD3/L-OHP cells. OCUM-2MD3/L-OHP cells were transfected with either HIF1α-siRNA or NS-siRNA (A), or transfected with 20, 40 or 80 nM of HIF1α-siRNA-2 (B), or transfected with 80 nM HIF1α-siRNA-2 for 24, 48 or 72 h (C), then cells were collected for Western blotting assays to calculate the knockdown efficiency. miR-27a levels were determined by qPCR (D). The survival rate of OCUM-2MD3/L-OHP was estimated by MTT assay.(E)Results were presented as the mean ± S.D. (n = 3). *P<0.01 compared with NS-siRNA group.

Western blot analysis showed that the mRNA and protein levels of HIF-1α decreased to varying degrees in OCUM-2MD3/L-OHP cells transfected with three pairs of HIF-1α-siRNA, whereas HIF-1α expression did not change in the cells transfected with control-siRNA. HIF-1α expression appeared to decline most steeply, by approximately 90%, using HIF-1α-siRNA-2 (Fig 3A). As shown in Fig 3B, HIF-1α expression decreased in these cells in a dose-dependent manner, in which HIF-1α expression decreased by more than 95% in cells transfected with 80 nM HIF-1α-siRNA-2, when HIF-1α-siRNA-2 was transfected further at a dose of 20 nM, 40 nM or 80 nM. In addition, the maximal inhibitory effect was detected 48 h after transfection (Fig 3C).

miR27a expression was significantly decreased following transfection of the OCUM-2 MD3/L-OHP cells with HIF-1α-siRNA-2 (Fig 3D). The MTT assay indicated that the cell survival rate was significantly reduced following treatment of HIF-1α-siRNA-2-transfected OCUM-2 MD3/L-OHP cells with L-OHP compared to non-transfected cells (Fig 3E).

### 4 Effects of miR27a on the MDR of OCUM-2MD3/L-OHP cells

**Fig 4:**
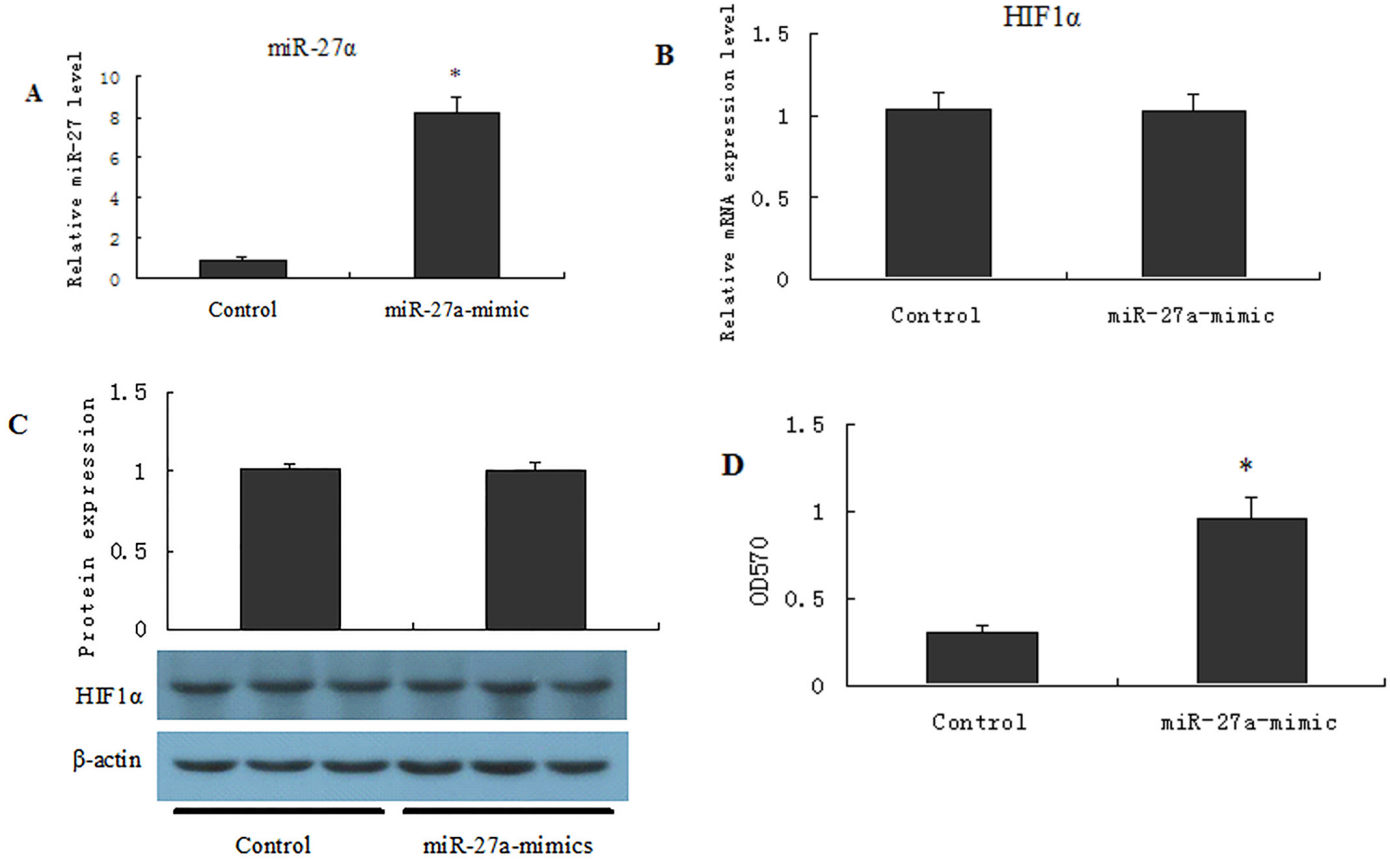
Effects of miR27a on MDR of drug resistant of OCUM-2MD3/L-OHP cell. OCUM-2MD3/L-OHP cells were transfected with miR27a analogues for 48 h (A), then cells were collected, miR27a and HIF1α levels were detected by RT-qPCR and Western blot assays (A, B and C). The survival rate of OCUM-2MD3/L-OHP was calculated by MTT assay (D). Results were presented as the mean ± S.D. (n = 3). *P<0.01 compared with control group.

The expression of miR27a was up-regulated in OCUM-2MD3/L-OHP cells when the miR27a mimic was co-transfected into these drug-resistant GC cells which had been transfected with HIF-1α-siRNA (Fig 4A). However, no significant difference in the expression of HIF-1α was detected in OCUM-2MD3/L-OHP cells after transfection (Fig 4B and 4C, qPCR and Western blot).

The MTT assay demonstrated that the survival rate of OCUM-2MD3/L-OHP cells was clearly increased following transfection with the miR27a mimic (Fig 4D, histogram results).

### 5 HIF-1α induces the transcription of miR27a in OCUM-2MD3 cells

**Fig 5:**
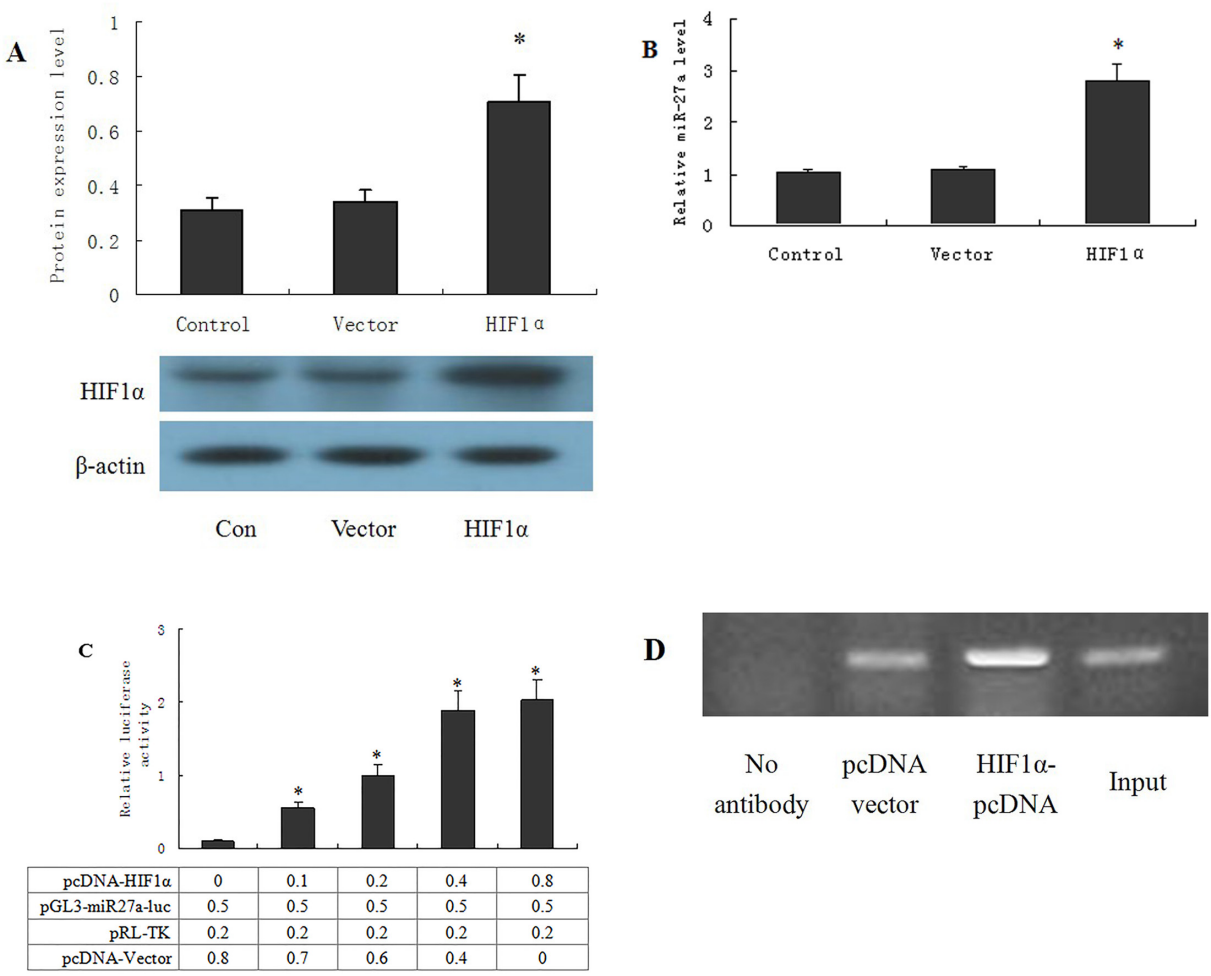
HIF-1α induces the transcription of miR27a in OCUM-2MD3 cells. OCUM-2MD3 cells were transfected with pcDNA-HIF-1α, the level of HIF-1α and miR27a were determined by Western blot and RT-qPCR (A and B) after 48h of transfection. Dual luciferase reporter assay was performed by cotransfection of the reporter plasmid containing the miR27a promoter and pcDNA-HIF-1αin OCUM-2MD3 cells(C), and ChIP assay was performed to test the binding of HIF-1α to the promoter of miR27a in OCUM-2MD3 cells. Results were presented as the mean ± S.D. (n = 3). *P<0.01 compared with pcDNA empty vector control group.

The expression of HIF-1α was significantly increased in OCUM-2MD3 cells transfected with the eukaryotic expression plasmid pcDNA-HIF-1α for 48 h (Fig 5A). Furthermore, miR27a expression was clearly up-regulated (Fig 5B). Thus, these results suggested that HIF-1α is a critical factor that affects the transcription of miR27a. Therefore, we established a luciferase reporter gene plasmid carrying a 2 kb sequence upstream of the promoter region of miR27a, which was co-transfected with pcDNA-HIF-1α into the cells. The DLA data showed that HIF-1α enhanced the promoter activity of miR27a following co-transfection (Fig 5C). ChIP analysis further confirmed that HIF-1α directly bound to the promoter region of miR27a (Fig 5D). The results indicated that HIF-1α may promote the transcription of miR27a in OCUM-2MD3 cells by directly binding to the promoter region of miR27a.

### 6 Inhibition of HIF-1α using siRNA suppresses the expression of drug resistance-related genes

**Fig 6:**
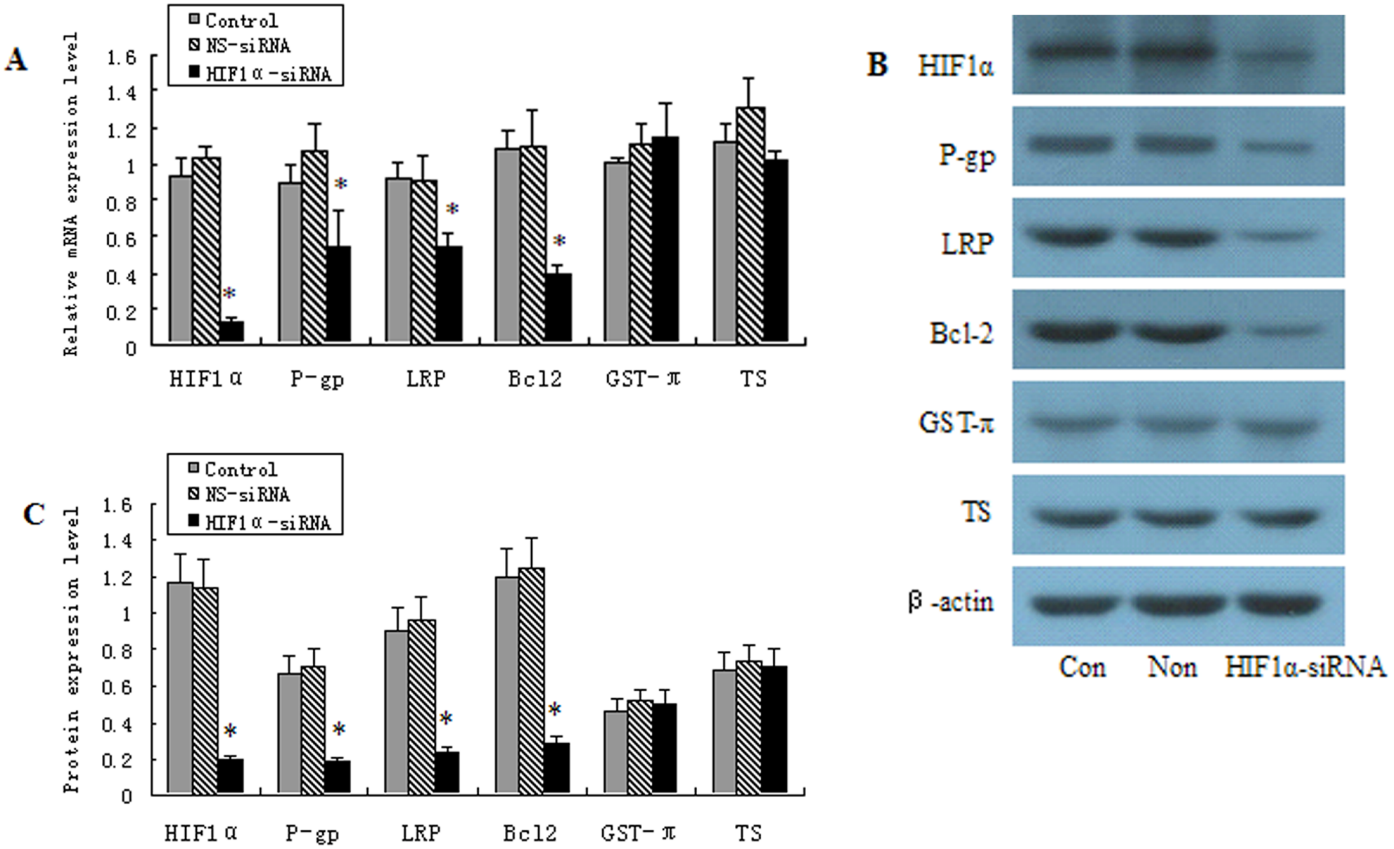
Inhibition of HIFα causes a reduction of drug resistance gene expression in OCUM-2MD3/L-OHP cells. OCUM-2MD3/L-OHP cells were transfected with 80 nM of HIF1α-siRNA or non-targeted siRNA or only treated with Lipofectamine 2000 for 48 h, then were subjected to RT-qPCR (A) and Western blotting (B, C) to detect the expression levels of MDR1/P-gp, LRP, Bcl-, GST-π and TS. β-actin was used for an endogenous reference to standardize the protein expression levels. Results were presented as the mean ± S.D. (n = 3). *P<0.01 compared with non-targeted siRNA group.

As shown in Fig 6, the inhibition of HIF-1α dramatically suppressed the expression of MDR1/P-gp, LRP, and Bcl-2 in OCUM-2MD3/L-OHP cells but did not significantly alter the expression of GST-π or TS. (Fig 6).

### 7 Inhibition of miR27a reduces of drug resistance-related gene expression in OCUM-2MD3/L-OHP cells

**Fig 7:**
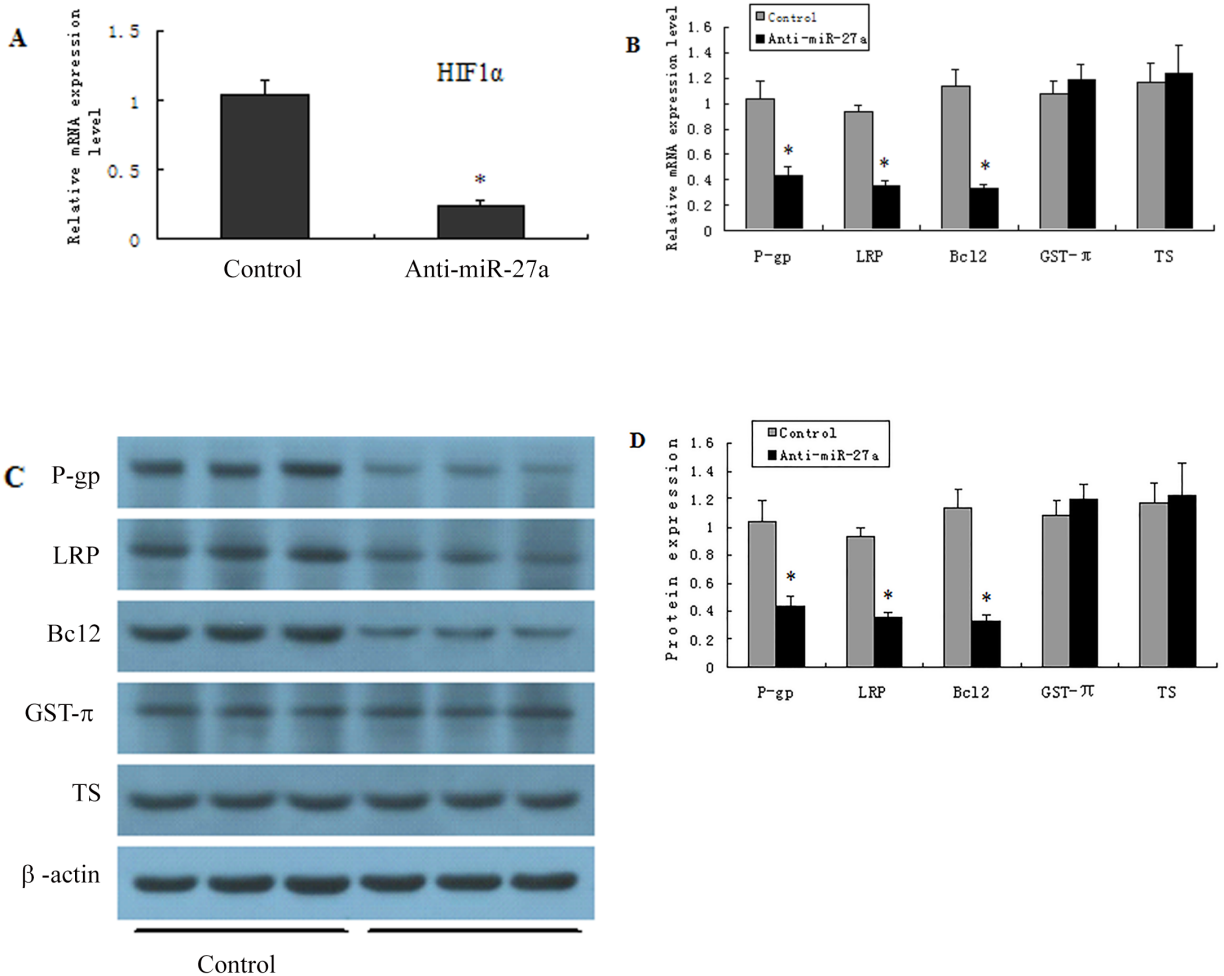
Inhibition of miR27a causes a reduction of drug resistance genes in OCUM-2MD3/L-OHP cells. OCUM-2MD3/L-OHP cells were transfected with Anti-miR27a (A), the expression levels of MDR1/P-gp, LRP, Bcl-, GST-π and TS were detected by RT-qPCR (B) and Western blotting (C, D). β-actin was used for an endogenous reference to standardize the protein expression levels. Results were presented as the mean ± S.D. (n = 3). *P<0.01 compared with non-targeted siRNA group.

miR27a was repressed in drug-resistant GC OCUM-2MD3/L-OHP cells transfected with the anti-miR27a sequence (Fig 7A). Furthermore, following this transfection, the expression of MDR1/P-gp, LRP and Bcl-2 was significantly decreased, whereas no significant difference was detected in the expression of GST-π or TS (Fig 7B, 7C and 7D, qPCR and Western blot). (Fig 7)

## Discussion

Although the worldwide incidence rate of GC appears to have decreased in recent years, a high incidence of GC persists, seriously endangering the health of individuals in Asia [22]. The MDR of GC cells contributes to the poor prognosis of GC, in which oxygen deficiency plays a critical role. In the present study, we established the stable OCUM-2MD3/L-OHP cell line that is resistant to L-OHP. Our data indicated that GC tissues and cell lines exhibit stronger drug resistance than normal gastric epithelial tissues and cell lines. We also found that drug-resistant cells develop much stronger MDR. Our results showed increased expression of HIF-1α in GC tissues and cell lines, and the highest expression of HIF-1α was detected in a drug-resistant cell line. Therefore, we suggest that HIF-1α contributes to the development of MDR in GC cells.

The HIF-1α gene encodes a protein that consists of 826 amino acids with a molecular weight of 120 kDa [23]. Many studies have confirmed that increased expression of HIF-1α is strongly associated with the occurrence and development of tumors[24–28]. Other studies have suggested that over-expression of HIF-1α enhances the drug-resistant properties of a variety of tumor cells[29–32]. In addition, our study demonstrated that GC tissues and cell lines exhibit stronger resistance to chemotherapeutic drugs than gastric para-carcinoma tissues and gastric mucosa cell lines. We also detected the most potent drug-resistance in GC cells. However, the mechanisms by which HIF-1α regulates MDR have yet to be clearly identified in GC cells. Therefore, we further investigated the effects of HIF-1α on the MDR properties of GC cells via gene interference and cloning techniques. RNA interference (RNAi) has displayed advantages in specificity, efficiency and durability for the regulation of target gene expression [33]. Our data indicate that inhibition of HIF-1α significantly reduced drug resistance in OCUM-2MD3/L-OHP cells. Moreover, we demonstrated that drug resistance was dramatically increased when pcDNA-HIF-1α, which was used to overexpress HIF-1α, was transfected into non-drug-resistant GC cell lines. Our results indicated that HIF-1α plays an essential role in the development of MDR in GC.

Recent studies have indicated that MDR is closely related with miRNAs in tumors[34–36]. Hu has reported that inactivating miR-27a may reverse the MDR properties of GC cells [18]. Moreover, it has been reported that miR-21, miR-27a, miR-210 and miR-181b were up-regulated via the HIF pathway in the hypoxic environment based on a gene-chip assay [19]. In accordance with our results, HIF-1α was positively associated with the expression of miR-27a in GC tissues and cell lines, and inhibition of HIF-1α decreased miR-27a. In contrast, over-expression of HIF-1α induced the expression of miR-27a. These results indicated that HIF-1α regulates the expression of miR-27a. Furthermore, our study showed that miR-27a mimics rescued the drug-resistance properties of HIF-1α-knockdown cells. Most importantly, HIF-1α directly binds to and promotes miR27a transcription, indicating that HIF-1α regulates the MDR of GC via miR-27a.

We characterized the expression of genes that are closely associated with MDR, including MDR1/P-gp, GST-π, LRP, Bcl-2 and TS, before and after the modulation of HIF-1α expression in GC cells to elucidate the mechanism by which the HIF-1α-miR-27a pathway regulates the MDR of GC. We demonstrated that inhibition of HIF-1α expression reduced the expression of MDR1/P-gp, LRP and Bcl-2. The expression levels of these genes were significantly increased when the cells were transfected with the miR-27a mimic, while the expression levels of GST-π and TS were not significantly altered. In accordance with this finding, over-expression of HIF-1α potently up-regulated the expression of MDR1/P-gp, LRP, and Bcl-2 in the GC OCUM-2MD3 cell line. Therefore, our data demonstrate that the HIF-1α-miR-27a pathway mediates MDR properties in GC by inducing MDR1/P-gp, LRP and Bcl-2 expression.

Our studies demonstrate that HIF-1α and miR-27a are up-regulated in GC tissues and cell lines. HIF-1α acts as an upstream regulator of miR-27a. HIF-1α-miR-27a signaling enhances the properties of MDR by inducing the expression of MDR1/P-gp, LRP and Bcl-2 in GC. These results suggest that the HIF-1α-miR-27a pathway plays a crucial role in the initiation of MDR in human GC, which may serve as a novel therapeutic target for MDR in GC.

## Supporting Information

S1 File Data of MTT and Western. (ZIP)
